# Norms of prejudice: political identity and polarization

**DOI:** 10.1098/rstb.2023.0030

**Published:** 2024-03-11

**Authors:** Alvarez-Benjumea Amalia, Fabian Winter, Nan Zhang

**Affiliations:** ^1^ The Spanish National Research Council (CSIC), 28037 Madrid, Comunidad de Madrid, Spain; ^2^ Department of Sociology, University of Zürich, 8050 Zurich, Switzerland; ^3^ MZES, University of Mannheim, 68159 Mannheim, Baden-Württemberg, Germany; ^4^ Max Planck Institute for Research on Collective Goods, Kurt-Schumacher-Str. 10, 53113 Bonn, Germany

**Keywords:** demographic change, majority–minority shift, social norms, prejudice

## Abstract

The USA is fast becoming a ‘majority–minority’ country in which Whites will no longer comprise the numerically dominant racial group. Prior studies have linked Whites’ status decline to heightened in-group solidarity and the feeling that Whites, as a group, face growing discrimination. In the light of these findings, we examine the extent to which a social norm controlling *anti-White prejudice* is now discernible in the USA. Drawing from an original survey measuring Americans’ reactions to racially-offensive speech, we examine second-order beliefs about the social inappropriateness of offensive statements targeting White Americans. We find that White Americans (in comparison to non-Whites) are indeed more likely to profess a social norm governing anti-white prejudice. The pattern is most discernible among white Republicans whom we expect to be most fearful of demographic change.

This article is part of the theme issue ‘Social norm change: drivers and consequences’.

## 1. Introduction

Sometime around the year 2040, the USA is projected to become a ‘majority–minority’ country in which Whites will no longer comprise the numerically dominant racial group [1]. This demographic shift has already profoundly impacted racial attitudes and intergroup relations. The erosion of Whites’ numerical, political and socio-economic status has been linked to feelings of group threat, a stronger sense of White in-group identity and the belief that Whites, as a group, increasingly face discrimination in American society [2–4].

In the light of these developments, we examine whether a social norm controlling *anti-White prejudice* is now discernible in the USA. Certain social groups are perceived as more socially acceptable targets of prejudice than others. People typically feel comfortable expressing offensive views on, for example, wealthy individuals, but they would refrain from expressing prejudice against ethnic minorities. The definition of what is considered an inappropriate target of prejudice can change over time, and is context-dependent [5,6].

Traditionally, scholars have focused on norms around anti-minority prejudice [7,8], while largely overlooking the social acceptability of prejudice directed against Whites. This lack of attention has largely reflected a social reality where Whites, as the dominant group, are less likely to suffer disadvantages owing to their race. In fact, for most of US history, Whites had little reason to think of themselves as members of a distinct racial group given their defining position within the American ‘mainstream’ [3, pp. 35–36]. That said, questions surrounding anti-White prejudice take on new significance in the wake current demographic shifts. More specifically, when Whites feel threatened and discriminated against, and they perceive more strongly that they share a ‘common fate’ with other Whites, conditions may be ripe for a social norm against anti-White prejudice to emerge.

We examine this proposition using data from a large-scale original survey measuring Americans’ reactions to racially-offensive speech. Our survey focuses on responses to a large set of ‘naturally occurring’ statements that include both subtle and extreme expressions of prejudice targeting a wide range of groups. Importantly, we ask respondents to rate the offensiveness of such statements in terms of both personal norms (i.e. how inappropriate they personally find such statements), as well as social norms (i.e. how such statements would be judged by most *other people*). Our analysis focuses on these second-order beliefs and compares Whites’ and non-Whites’ ratings of the social acceptability of anti-White statements. We also devote particular attention to the responses of White Republicans, whom we expect to (i) be more fearful of demographic change [1,9] and (ii) identify more strongly with their own race [3].

## 2. Method

Our study draws upon data from an original survey measuring Americans’ reactions to bigoted and prejudiced speech.^1^ Our survey was fielded between May and December 2020 using YouGov’s online access panel, from which over 5000 respondents were recruited on the basis of nationally representative gender, age, education, region and racial quotas. We designed the survey to capture responses to a wide range of contemporary expressions of prejudice targeting many different groups.

In the first step of our research, we recruited workers from Amazon Mechanical Turk to provide us with statements that they considered to be potentially offensive about 9 different groups: LGBTQ+, women, Hispanics, Blacks, Asians, Muslims, people with disabilities, the elderly, and Whites. From this list, we next selected subsets of approximately 20 statements pertaining to each group. We made an effort to include different types of prejudicial statements ranging from ‘micro-aggressions’ (e.g. ‘Wow, he’s really smart for a Black guy’) to explicit racial slurs. Thus the statements vary in their level of offensiveness. A full list of the statements we employed is provided in electronic supplementary material, appendix A1.

With our selection in hand, we next presented respondents in the YouGov panel with two different sets of randomly selected statements. In the first set, respondents were asked to indicate how inappropriate or offensive, if at all, they personally found each statement on a 4-point scale from ‘not at all offensive’ to ‘extremely offensive’. In the second set, respondents were instead asked to indicate how inappropriate or offensive *most people* would find each statement using the same 4-point scale.^2^ These second-order beliefs constitute our measure of the social norm with respect to prejudice towards the different groups.^3,4^

Our analysis dataset comprises a total of 79 021 ratings from 4579 respondents interviewed before the 2020 US Presidential Election.^5,6^ Summary statistics on the characteristics of this sample are presented in table 1, along with American Community Survey (ACS) benchmarks for comparison. We note that our analytic sample is somewhat more White, slightly older, and better educated than the ACS benchmarks, but is otherwise fairly comparable to the overall US population.

**Table 1.** Summary statistics: analytic sample. The analytic sample comprises 4579 respondents recruited from YouGov’s online panel. Demographic benchmarks are drawn from the 2019 American Community Survey (ACS).

		analytic sample (%)	ACS 2019 (%)
sex	male	46	48.9
	female	54	51.1
age	18–29	15.6	20.9
	30–44	25.2	25.1
	45–64	34.9	32.9
	65+	24.3	21.1
race	White	72	63.1
	Black	9.7	12.6
	hispanic	12	16.5
	Asian	3.7	6.3
	other	—	1.5
education	high school	30	38.1
	some college, 2-years	26	28.2
	4-years	24	21.7
	post-grad	20	12.0
region	northeast	19	17.4
	midwest	21.0	20.8
	south	40	38.0
	west	20	23.8

Racial identity was self-identified using the questions ‘What racial or ethnic group best describes you?’. Partisanship was coded from two questions asking (i) whether the respondent identifies as a Democrat or Republican and (ii) whether they lean towards one of the two parties. Democrat and Republican ‘leaners’ were counted as partisan identifiers. The third ‘non-affiliated’ category includes Independents with no partisan lean, respondents identifying with third parties and individuals who declined to provide a party affiliation (table 2).

**Table 2.** Partisanship breakdown of the analytic sample. *Note*. Analytic sample comprises 4579 respondents recruited from YouGov’s online panel.

		*N*	per cent
White (total)		3317	72
	Republican	1354	29
	Non-affiliated	592	13
	Democrat	1371	30
non-White		1280	28
sample total		4597	100

Each respondent in our YouGov poll rated 36 statements in total: 18 statements on the social norm scale and 18 statements on the personal norm scale.^7^ Our analyses focus on the subset of 8752 ratings measuring the social norm governing anti-White statements. A short selection of these statements is displayed below^8^:

Ex 1: *It must be so boring to hang out with White people all of the time...White people are so lame.*

Ex 2: *The world could get by just fine with zero White people.*

Ex 3: *White people have everything handed to them on a silver platter from birth.*

We standardize inappropriateness ratings within each statement, effectively zeroing out between-statement differences. Of course, with this procedure, we can no longer analyse whether some statements are seen as more offensive than others. However, comparisons *across statements* are *per se* difficult—for instance, there is no ‘equivalent’ of the *n*-word that could be applied to other groups. Consequently, rather than compare across statements, our analytical strategy focuses on differences *across respondents* when rating the same statements. To give a specific example, our analysis asks: when presented with the statement ‘The world could get by just fine with zero White people,’ are Whites more or less likely than non-Whites to judge the statement as socially inappropriate?

## 3. Results

**Figure 1.**
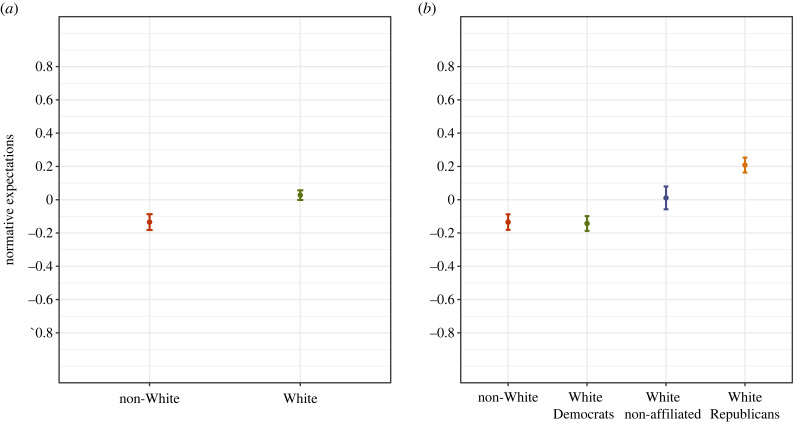
Differences in second-order beliefs about anti-White statements. The figure displays the predicted values of second-order beliefs provided by White Republicans, White Democrats, Non-Affiliated Whites and Non-Whites irrespective of self-identified party affiliation. Second-order beliefs are captured in a survey item where respondents rated: ‘How would MOST PEOPLE react to [statement]’. Greater (standardized) scores indicate higher expected disapproval. Results come from a multilevel regression model with a random intercept for respondents and a random intercept for statements. The full model is reported in electronic supplementary material, appendix table A1. *N* = 8752 statement ratings provided by 4512 participants. The coefficients are depicted as dots; 95% confidence intervals are indicated by the ends of the vertical error bars. (Online version in colour.)

To address this question, we estimate a model regressing (standardized) social norm ratings of anti-White statements on an indicator variable for White (versus non-White) respondents. Our models include random intercepts at the level of both participants and statements. Marginal means are plotted in figure 1*a* and the full model is reported in Model 1 of electronic supplementary material, appendix A1. Higher values represent more socially inappropriate ratings. We observe that, on average, Whites (in comparison to non-Whites) rate anti-White statements as approximately 0.16 standard units more ‘socially inappropriate’ (s.e. = 0.03, *p*-value < 0.01). These results change very little with the inclusion of controls for gender, age, education, employment status and region of residence (see Model 2 of electronic supplementary material, appendix table A1). In short, it appears that a stronger social norm against anti-White prejudice exists for Whites (in comparison to non-Whites).

To dig deeper into these results, we next disaggregate White respondents by partisan affiliation. Given prior research showing that White Republicans in particular are most threatened by demographic change [2–4], we re-estimate our baseline model with a series of indicator variables for White Republicans, White Democrats, Non-Affiliated Whites and Non-Whites.

Marginal means are plotted in figure 1*b* and the full model is reported in Model 3 of electronic supplementary material, appendix table A1.

We observe that the main differences between Whites and non-Whites are driven by the second-order beliefs of White Republicans. More specifically, White Democrats are virtually indistinguishable from non-Whites in their perceptions of the social norm (*β* = −0.01, s.e. = 0.03, *p*-value = n.s.). And while there is some indication that politically non-affiliated Whites perceive anti-White statements as more socially inappropriate than non-Whites (*β* = 0.15, s.e. = 0.04, *p*-value < 0.01), the gap between non-Whites and White Republicans is over twice as large (*β* = 0.36, s.e. = 0.03, *p*-value < 0.01). These results change very little with the inclusion of controls for gender, age, education, employment status and region of residence (see Model 4 of electronic supplementary material, appendix table A1).^9^

To benchmark the substantive significance of these results, we compared White Republicans against the rest of the YouGov sample when rating the social appropriateness of the full set of prejudicial statements (i.e. targeting racial, sexual and other minorities). Here it should be noted that our data were collected in the late summer/autumn of 2020, in a political climate where the Republican standard-bearer Donald Trump had become notorious for his incendiary statements targeting (Hispanic) immigrants and Muslims. Indeed, political commentators have linked Trump’s provocative rhetoric to a more general erosion of social norms protecting ethnic/racial minorities amongst his Republican base [5,12–14]. Thus it would be informative to compare White Republicans’ perceptions of the norm governing anti-White prejudice against other more-established taboos.

**Figure 2.**
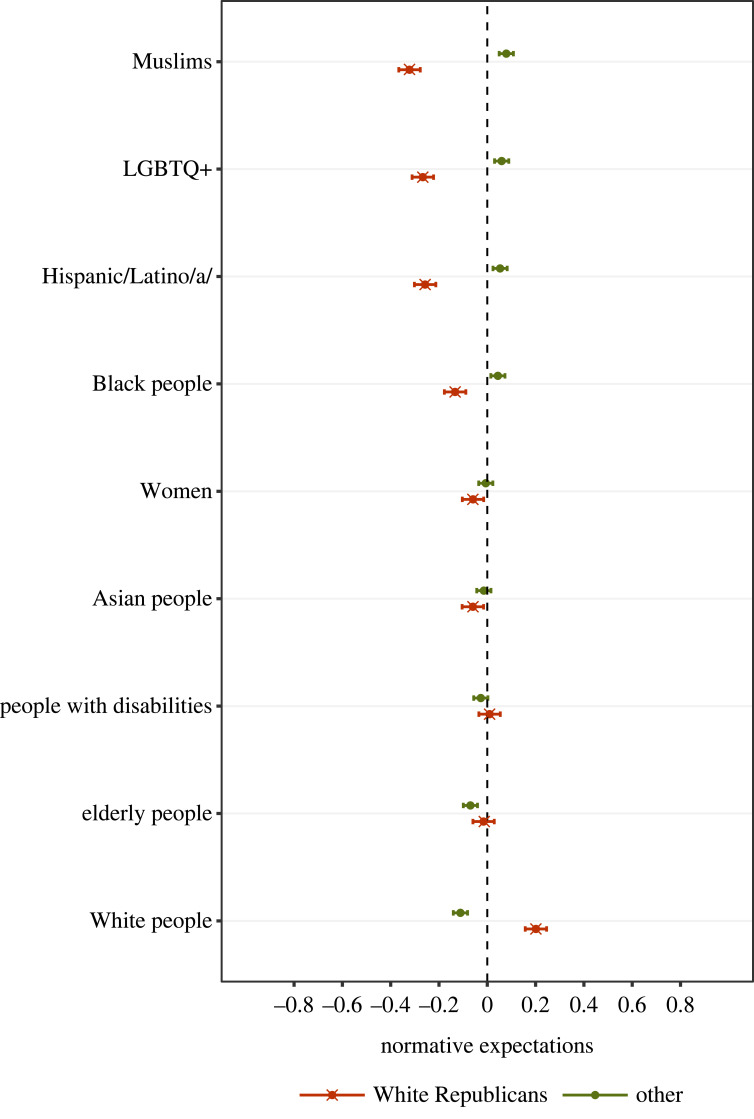
Differences in second-order beliefs about statements targeting different groups. The figure displays the predicted values of second-order beliefs provided by White Republicans versus the rest of the sample. Second-order beliefs are captured in a survey item where respondents rated: ‘How would MOST PEOPLE react to [statement]’. Greater (standardized) scores indicate higher expected disapproval. Results come from a multilevel regression model with a random intercept for participants and a random intercept for statements. The full model is reported in electronic supplementary material, appendix table A2. *N* = 79 021 statement ratings provided by 4597 participants. The coefficients are depicted as dots; 95% confidence intervals are indicated by the ends of the horizontal error bars. (Online version in colour.)

To do so, we used the full set of 79 021 social norm ratings and re-estimated our baseline model with an interaction term between White Republicans × statement target, where statement target denotes a set of dummy variables for White target, Black target, elderly target, etc. Marginal means are plotted in figure 2 and the full model results are presented electronic supplementary material, appendix table A2. We observe very small differences between White Republicans and other respondents in perceptions of the social norm against prejudice targeting the elderly, people with disabilities, Asians and women. For anti-Black statements, White Republicans appear to perceive a ‘looser’ norm than the rest of the sample (*β* = −0.17, s.e. = 0.03, *p*-value < 0.001), but the absolute value of the difference is about half as large as the gap with respect to anti-White statements (*β* = 0.31, s.e. = 0.03, *p*-value < 0.001). It is only when we come to statements targeting Hispanics, the LGBTQ+ community and Muslims that we observe similarly large levels of normative polarization (but in the other direction). In other words, it appears that White Republicans are about as *restrictive* (relative to the remainder of the sample) of speech targeting fellow Whites as they are *permissive* of speech targeting Muslims, Hispanics and the LGBTQ+ community.

**Figure 3.**
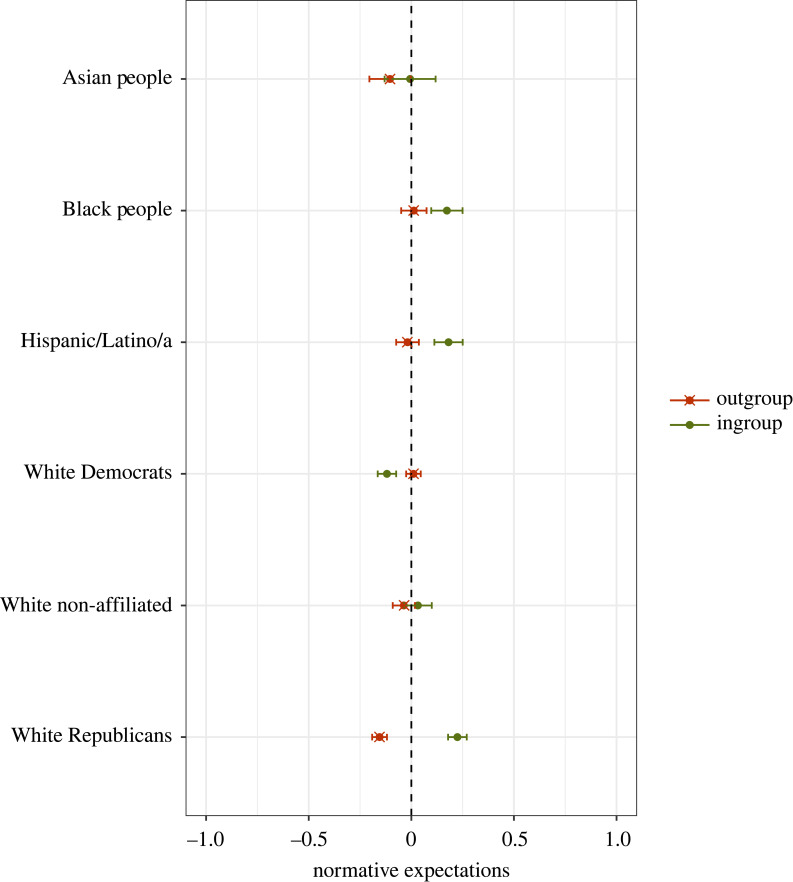
Differences in second-order beliefs about statements targeting Asian people, Black people, hispanic/latino/a, and White people. The figure displays the predicted values of second-order beliefs about ingroup versus outgroup targets. Second-order beliefs are captured in a survey item where respondents rated: ‘How would MOST PEOPLE react to [statement]’. Greater (standardized) scores indicate higher expected disapproval. Results come from a multilevel regression model with a random intercept for participants and a random intercept for statements. The full model is reported in electronic supplementary material, appendix table A3. *N* = 34 311 statement ratings provided by 4472 participants. The coefficients are depicted as dots; 95% confidence intervals are indicated by the ends of the horizontal error bars. (Online version in colour.)

To some extent, the preceding results could also be interpreted as a form of ‘ingroup favouritism’ on the part of White Republicans regarding (perceived) social norms governing anti-White prejudice. Along these lines, a second useful benchmark would be to compare the level of *normative ingroup favoritism* amongst White Republicans versus other Respondent groups. Here, we focus on Asian, Black and Hispanic respondents, as well as White Democrats and non-affiliated Whites. We use a subset of 34 311 ratings (pertaining to racial groupings only) and estimate a regression model that includes an interaction term between Respondent group × ingroup, where ingroup is an indicator variable that assumes a value of 1 when a statement was assessed by members of the target group (e.g. anti-Black comments rated by Black participants). The predicted values are plotted in figure 3. The relevant coefficients and full results are presented in electronic supplementary material, appendix table A3.

We observe that Blacks, Latinos and White Republicans tend to rate statements targeting their own group as more socially inappropriate than statements targeting outgroups. What is striking, however, is that this ‘double standard’ is most pronounced amongst White Republicans (*β* = 0.38, s.e. = 0.02, *p*-value < 0.001). For context, the effect observed for White Republicans is almost twice that of the effect observed for Hispanics (*β* = 0.20, s.e. = 0.03, *p*-value < 0.001) and more than two times larger than the effect for Black people (*β* = 0.16, s.e. = 0.03, *p*-value < 0.001). Finally, it is interesting that White Democrats display the opposite pattern and apply a ‘looser’ norm to anti-White statements than to statements about other groups (*β* = −0.13, s.e. = 0.02, *p*-value < 0.001).

## 4. Discussion

Taken together, the prior analyses point to a strong norm against anti-White prejudice among White Republicans. Prior research argues that this norm has only *recently emerged* in response to growing fears about demographic change [2–4]. Although we believe that our results lend support to these arguments, we acknowledge that our cross-sectional data cannot directly capture this dynamic processes.

**Figure 4.**
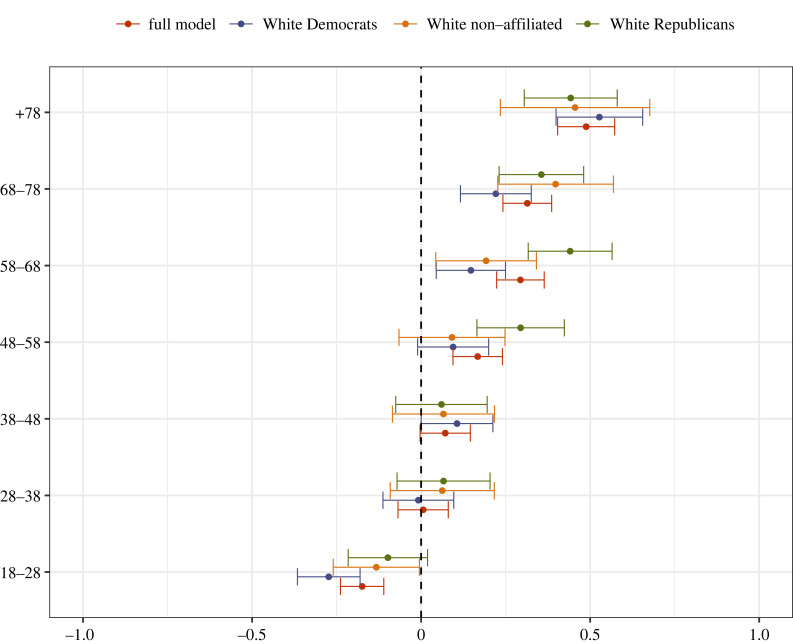
Differences in second-order beliefs about anti-White statements by Age. The figure displays coefficients of second-order beliefs provided by all Whites, White Republicans, White Democrats and Non-Affiliated Whites. Second-order beliefs are captured in a survey item where respondents rated: ‘How would MOST PEOPLE react to [statement]’. Greater (standardized) scores indicate higher expected disapproval. *N* = 8752 statement ratings provided by 4512 participants. Results come from a multilevel regression model with a random intercept for respondents and a random intercept for statements. The full model is reported in electronic supplementary material, appendix table A4. The predicted values are depicted as dots; 95% confidence intervals are indicated by the ends of the horizontal error bars.

As an alternative, we provide two supplementary analyses that would be consistent with a dynamic account.^10^ First, we compare patterns across age cohorts, under the assumption that demographic changes are more threatening for older individuals who were socialized during a less diverse era in American history. We may therefore expect older Whites to rate anti-White statements as more socially inappropriate (compared to younger Whites). Indeed, as shown in figure 4, this is exactly the pattern that emerges.^11^

**Figure 5.**
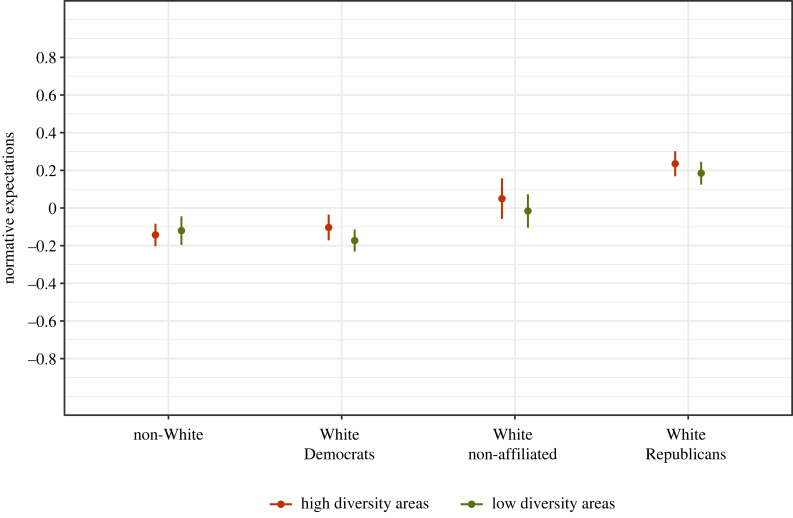
Differences in second-order beliefs about anti-White statements. The figure displays the predicted values of second-order beliefs provided by White Republicans, White Democrats, Non-Affiliated Whites and Non-Whites irrespective of self-identified party affiliation, separately for respondents living in ‘high diversity’ versus ‘low diversity’ states. Second-order beliefs are captured in a survey item where respondents rated: ‘How would MOST PEOPLE react to [statement]’. Greater (standardized) scores indicate higher expected disapproval. *N* = 8752 statement ratings were provided by 4512 participants. Results come from two multilevel regression models with a random intercept for respondents and a random intercept for statements. The full models are reported in electronic supplementary material, appendix table A5. The predicted values are depicted as dots; 95% confidence intervals are indicated by the ends of the horizontal error bars. (Online version in colour.)

Secondly, we compare patterns across US states that have experienced demographic changes to differing degrees, under the assumption that the norm would be stronger in states where Whites comprise a smaller share of the population (and hence feel more racially threatened [9]). For this analysis, we first divided states using median split (the median White population proportion in the US is approx. 61%^12^) and replicated our main analyses from figure 1 separately for individuals residing in ‘high diversity’ versus ‘low diversity’ areas.^13^ As shown in figure 5, the patterns are indeed more pronounced for White respondents living in more diverse states. Taken together, figures 4 and 5 provide additional evidence consistent with a dynamic process of normative change.

## 5. Conclusion

The USA is in the midst of an unprecedented demographic transition wherein Whites will soon become a numerical minority within American society. Political commentators have linked this impending loss of status to both increased racial threat as well as a stronger sense of White identity [2,3,9]. Along these lines, the present study demonstrates that Whites (in comparison to other Americans) are also more likely to perceive a social norm governing anti-White prejudice. Interestingly, the strength of this norm follows a political ‘gradient’ and is most pronounced among White Republicans.

Within this context, an interesting question is whether this norm can be expected to spread outside the core of White Republicans? For instance, it is possible that *racial minorities* within the Republican party will come to adopt the same position as White Republicans on the social inappropriateness of anti-White prejudice (in much the same way that White Democrats have come to adopt norms around anti-minority prejudice). At the same time, the social norm appears strongly linked to the Republican party. And given current levels of affective polarization in American society [15], efforts at norm propagation will likely make little headway among Democrats of any race. These considerations therefore suggest that the norm against anti-White prejudice is likely to remain a ‘particularistic norm’ for some time to come.

## Data Availability

The data are publicly available from the OSF repository: https://osf.io/j8mds/ [16]. The data are also provided in electronic supplementary material [17].

## Endnotes

^1^
IRB approval for this project was obtained from the University of Bonn Ethics Commission. Lfd. no. 417/19.

^2^
Respondents were told: ‘In the following screens, you will again be asked to read a series of statements. However, we would now like you to set aside your personal feelings towards the use of such language. Instead, please tell us how you think most Americans would react to these statements’. In order to reduce social desirability, we also explicitly reminded respondents that some people may consider these statements to be inappropriate or offensive, while others may find such language to be more or less acceptable. Each respondent rated 36 statements in total. Importantly, the different statements were used to elicit personal norms (first-order beliefs) and social norms (second-order beliefs)—i.e. no statement was rated twice by the same respondent.

^3^
This elicitation task was non-incentivized. Past research shows that people provide similar responses in incentivized and non-incentivized elicitation tasks [10].

^4^
The data are publicly available at https://osf.io/j8mds/.

^5^
We also fielded a small number of survey waves after the 2020 Election that included some re-interviews of prior participants. We reserve analyses of these data for a separate paper.

^6^
We dropped individuals who provided ‘straight-line’ survey responses (i.e. respondents who rated all statements with the same rating), as well as those individuals who indicated that they provided non-serious answers to survey questions ‘always’ or ‘most of the time’ [11].

^7^
In practice, there were a small number (less than 5%) of missing values where individuals did not rate a particular statement.

^8^
The full set of statements is provided in electronic supplementary material, appendix §A1.

^9^
We conducted further analyses to explore whether the norm applies to prejudiced comments about White people in general, or only to specific forms of anti-White prejudice. In particular, we categorized three versions of anti-White statements: (i) assertions that ‘White people are all racist’; (ii) denials that Whites can be the victims of prejudice; and (iii) a residual category comprising miscellaneous negative comments about Whites. To determine whether a particular category of statements may be driving our results, we ran a series of models similar to those presented in figure 1*b* where we sequentially excluded categories (i) or (ii), and both at the same time. Results are shown in electronic supplementary material, appendix table A6. We found virtually identical patterns to those reported in figure 1*b* across all sequential exclusions, indicating that no single ‘type’ of anti-White prejudice is driving our results.

^10^
We thank an anonymous reviewer for these suggestions.

^11^
The full models are reported in electronic supplementary material, appendix table A4.

^12^
These data are taken from https://www.census.gov/library/visualizations/interactive/race-and-ethnicity-in-the-united-state-2010-and-2020-census.html.

^13^
The full models are reported in electronic supplementary material, appendix table A5.
